# Smartphone addiction and associated factors among postgraduate students in an Arabic sample: a cross-sectional study

**DOI:** 10.1186/s12888-021-03285-0

**Published:** 2021-06-10

**Authors:** Asem A. Alageel, Rayyan A. Alyahya, Yasser A. Bahatheq, Norah A. Alzunaydi, Raed A. Alghamdi, Nader M Alrahili, Roger S. McIntyre, Michelle Iacobucci

**Affiliations:** 1 Assistant Professor of Psychiatry, Department of Clinical Neurosciences, College of Medicine, Al-Imam Muhammad Ibn Saud Islamic University (IMSIU), P.O. Box 26181, Riyadh, 11486 Saudi Arabia; 2 Collage of Medicine, Al-Imam Muhammad Ibn Saud Islamic University (IMSIU), Riyadh, Saudi Arabia; 3 Prince Sultan Military Medical City (PSMMC), Riyadh, Saudi Arabia; 4 Department of Clinical Neurosciences, College of Medicine, Al Imam Mohammad ibn Saud Islamic University (IMSIU), Riyadh, Saudi Arabia; 5 Mood Disorders Psychopharmacology Unit, University Health Network, Toronto, ON Canada

**Keywords:** ADHD, Depression, Insomnia, Postgraduate, Smartphone, Smoking

## Abstract

**Background:**

Smartphone addiction, as with other behavioral addictions, is associated with social, physical, and mental health issues. In this article, we investigated the prevalence of smartphone addiction among postgraduate students and evaluated its correlation with social demographics, depression, attention-deficit/hyperactivity disorder (ADHD), and nicotine dependence.

**Objectives:**

The objective of this study was to investigate the prevalence of smartphone addiction among Middle Eastern postgraduate students, determine the factors associated with smartphone addiction, and estimate the incidence rate of major depressive disorder (MDD), ADHD, insomnia, and nicotine addiction among postgraduate students with smartphone addiction.

**Methods:**

As part of a cross-sectional online survey, participants were given a self-questionnaire divided into six sections: Socio-demographics, Smartphone Addiction Scale (SAS), Patient Health Questionnaire (PHQ9) for Depression, Athens Insomnia Scale (AIS), the Fagerström Test for Cigarette Dependence Questionnaire (FTCd), and the adult ADHD Self-Report Scale (ASRS-v1.1).

**Results:**

Of the 506 patients, 51.0% of the participants demonstrated smartphone addiction. A significant association was also observed between extensive smartphone use and MDD (*P* = 0.001). Of the smokers in this study, 41.5% were addicted to smartphones (*P* = 0.039). Smartphone addicts had approximately two times the chance of having insomnia (OR = 2.113) (*P* = 0.013). In addition, they showcased more ADHD symptoms (OR = 2.712) (*P* < 0.001).

**Conclusions:**

We found a positive association among insomnia, depression, adult ADHD, and smartphone addiction, which confirms the findings reported in the previous studies. Therefore, we encourage the scientific community to further study the impacts of smartphone addiction on the mental health of postgraduate students.

## Background

Smartphones are handheld mobile devices with many convenient features and software applications (email, social media, web browser, etc.), which are operated via an Internet connection. The first smartphone was produced in 1992, but the term “smartphone” was designated in 1995, when smartphone functions evolved to include more than communications. Currently, smartphones provide entertainment, social media, health monitoring, productivity, utility functions (e.g., day planners), text talk, photo editing, and many more features in one handheld device. With this wide array of functionalities built into smartphones, researchers have observed an increasing number of smartphone users. In 2017, Google announced that they had reached 2 billion active users; in 2019, this number reached 2.5 billion [1]. Additionally, in 2019, Apple announced 900 million active users [2]. In 2019, Google and Apple collectively announced that 3.4 billion people use Apple or Google smartphones. These numbers do not include people who are not using Apple or Google products.

According to the American Psychiatric Association (APA), addiction is “A complex condition, a brain disease that is manifested by compulsive substance use despite harmful consequences.” [3] Regardless of whether addiction is substance or behavior related, there are five elements of addiction [4]. The first element is feeling different; it includes the feeling of uncomfortability, loneliness, restlessness, or incompleteness [5]. The second element of addiction is a preoccupation with behavior; excessive thoughts about and desire to perform a behavior; excessive time spent planning and engaging in the behavior, including recovering from its effects; and less time spent on other activities [6], despite potentially diminishing appetitive effects [7, 8]. Temporary satiation is the third element of addiction; after acute engagement in addictive behavior, some period of time may occur in which urges are not operative, the addiction craving is “shut down,” but soon returns [9–11]. The fourth element is loss of control, wherein many people who claim to be struggling with addiction experience feeling obliged to exhibit addiction, which is associated with an experience of loss of control and, in some cases, neglect of essential self-care, which suggests a loss of will [12]. The final element is negative consequences, which involve ongoing engagement in addictive behavior despite suffering from numerous negative consequences. This last component of addiction has often been used as a criterion for identifying dependence on the addictive behavior [13].

“Smartphone addiction” is a form of technological addiction. Generally, it is similar to internet addiction. Smartphone addiction consists of four main components: compulsive behaviors, tolerance, withdrawal, and functional impairment [14]. In a study of 2367 university students in Riyadh, the results indicated that 27.2% of participants stated that they spent more than 8 h per day using their smartphones [15]. In another study conducted on 688 Lebanese university students, 49% reported excessive smartphone use (≥5 h/weekday) [16].

Major depressive disorder (MDD) is a mental illness characterized by debilitating changes in the way enthusiasm are felt when partaking in activities that the individual once enjoyed is a disturbance in cognitive functions, emotional regulation, affect processing, reward function, and circadian rhythms. MDD can manifest in a wide variety of symptoms, including lack of appetite, fatigue, trouble sleeping (e.g., insomnia), feelings of guilt, and thoughts of suicide. Depending on the severity, MDD can be associated with a degree of cognitive dysfunction that influences the ability to perform everyday home and work activities, causing various physical and emotional issues [3, 17, 18]. MDD seems to be closely associated with addiction and substance abuse. Two epidemiological studies in 1990 and 1994 provided evidence that mood disorders increase the risk of substance use disorders (SUD) [19, 20]. One literature review studied the relationship between alcohol use disorders (AUD) and MDD and found a correlation between the two, in that having AUD doubled the risk of developing MDD, and vice versa [21]. Mood disorders and SUD comorbidity lower the prognosis and treatment outcomes for each problem [22]. However, there is evidence to suggest that successful treatment of a comorbid mood disorder would decrease cravings and substance abuse [23]. Furthermore, the correlations are not exclusive to substance addiction, and several studies have concluded that behavioral addictions (such as Internet and smartphone addiction) can be associated with MDD [24, 25].

Insomnia is defined as a subjective perception of difficulty falling or staying asleep. It can have acute episodes lasting one night or chronically up to several weeks or months. It is associated with decreased mental and physical health-related quality of life scores [26] and psychiatric illness [27]. Furthermore, it is indirectly associated with smartphone overuse [28].

Attention-deficit hyperactivity disorder (ADHD) is a neurodevelopmental disorder usually diagnosed in childhood and may last into adulthood. It is characterized by hyperactivity, impulsiveness, or inattentiveness, and often all three symptoms (DSM-IV-TR; APA, 2000), which interfere with or affect the quality of social, academic, or occupational performance or development [29]. A study in different countries in America, Europe, and the Middle East demonstrated that the average adult ADHD prevalence was 3.4%, with a higher percentage in high-income countries (4.2%) compared with low-income countries (1.9%) [30]. Adults with ADHD have a significantly high chance of suffering from depression, antisocial personality, anxiety, and SUD [31].

Past studies on the prevalence of smartphone addiction and its relationship to mental and physical issues have been conducted [15, 16, 25, 32, 33]. These investigations revealed some of the components of smartphone addiction [28, 34–36]. Contrarily, numerous elements, such as ADHD or nicotine addiction, were left uninvestigated. The objective of this study was to discover the correlation between smartphone addiction and different elements, including MDD, nicotine dependence, quality of life, and sleep, in order to determine standard variables among postgraduates. Identifying these features of smartphone addiction can promote awareness and knowledge about smartphone addiction while surveying the level of its impact on mental health.

This study aimed to distinguish the prevalence of smartphone addiction among postgraduate students. Due to the high level of pressure experienced by postgraduate students [37], we assumed that they are especially vulnerable to smartphone addiction. Postgraduate students use smartphones for communication, research for school assignments, and entertainment. As far as we know, there have been no investigations on the prevalence of smartphone addiction among Arabian Middle Eastern postgraduate students. In this article, we will investigate the prevalence of smartphone addiction among postgraduate students and evaluate its correlation with social demographics, depression, ADHD, and nicotine dependence.

## Methods

A cross-sectional online survey was sent via email and social media accounts for postgraduate education (Twitter, Facebook, and WhatsApp) to post-secondary students and was completed by 558 participants. Participants were included in the study if they were Arabic speaking postgraduate students and smartphone users. Postgraduate students from 187 different universities participated in the study. The participants were studying in different countries worldwide, including Saudi Arabia, Jordan, Egypt, Kuwait, Algeria, Bahrain, Iraq, Lebanon, Afghanistan, Ethiopia, Fiji, Cyprus, Australia, England, United States, and Canada. We excluded 52 students from the study due to incomplete questionnaires, leaving us with a total of 506 participants, 385 (75..9%) of which were located in Saudi Arabia.

This study was approved by the Institutional Review Board (IRB) of Imam Mohammad Ibn Saud Islamic University in Riyadh, Saudi Arabia. Informed consent was obtained from all participants through a statement of agreement at the beginning of each questionnaire. All methods were thoroughly explained to each participant and performed in accordance with the relevant guidelines and regulations from the World Medical Association (WMA) Declaration of Helsinki.

The online survey consisted of 43 questions, which took approximately 5 to 10 min to complete. The questionnaire was divided into six parts. The first part was concerned about sociodemographic information, such as age and gender. The second and third parts included Arabic-validated versions of the Smartphone Addiction Scale (SAS) [38] and the Patient Health Questionnaire (PHQ9) for Depression [39] respectively. The fourth part used the Athens Insomnia Scale (AIS) to evaluate sleep quality [40]. The fifth section was concerned about nicotine dependence and employed the Fagerström Test for Cigarette Dependence Questionnaire (FTCd) [41]. Finally, the sixth part implements the Adult ADHD Self-Report Scale (ASRS-v1.1) [20].

Smartphone addiction was measured using the Arabic version of the Smartphone Addiction Scale (SAS). The SAS is a self-diagnosis scale modified from K-scale, which is a scale to evaluate Internet addiction (IA) in juveniles. The SAS consists of 33 items with 6 subscales, namely, daily life disturbance, positive anticipation, withdrawal, cyberspace-oriented relationship, overuse, and tolerance [42]. The items are scored on a six-point Likert scale: strongly disagree (1), disagree (2), weakly disagree (3), weakly agree (4), agree (5), or strongly agree (6). The sum of the six subscales refers to an SAS score with a range of 33 to 198. A higher score means higher addictive behavior using smartphones [42]. Data factorability for the Arabic version of the SAS was confirmed using the Kaiser–Meyer–Olkin (KMO) test of sampling adequacy with a resulting value of 0.94 and was supported with Bartlett’s test of sphericity to confirm the suitability of data for factor analysis, which demonstrated a significant value of *p* < 0.01 [38]. The internal consistency for the Arabic SAS was calculated using Cronbach’s alpha with a value of *α* = 0.94 [38]. In this study, we grouped the participants who scored 116 or more in SAS in the high smartphone use group, whereas the participants who scored less were placed in the low smartphone use group.

The second part of the questionnaire uses the PHQ9 for Depression, a self-report questionnaire designed to evaluate the level of depression over 2 weeks; a higher score indicates a higher chance of depression [43]. We used a validated and translated version to assess our Arabic population; it had an internal consistency reliability of 0.857, as calculated using Cronbach’s alpha [39]. We used a cut-off point of 10 for clinically significant depression and then further classified the depressed participants as clinically significant, moderately severe, or severe. We considered those who scored between 10 and 14 to have clinically significant depression, scores that ranged between 15 and 19 as moderately severe depression, and scores > 20 as severe depression. Furthermore, participants who scored 15+ warranted active treatment [43].

The AIS was adopted to measure sleep quality. The English version has an optimum specificity of 85% and sensitivity of 93% [40] and evaluates sleep quality over the last month using a four-point system of 0 to 3, where 0 means no insomnia symptoms and 3 means acute sleep difficulties. In our study, any participant with a score of 6 or more was considered to have insomnia. We used an Arabic version of AIS by the Toronto Sleep Clinic, which was translated to Arabic by an English-speaking healthcare professional whose mother tongue is Arabic. Another translator used the same approach to perform back-translation of the Arabic translation into English. A few English-speaking translators reviewed the back-translation for any problematic contextual discrepancies. Despite the use of measures to ensure accurate translation, the scale has yet to be tested for validity.

The fifth part employed the FTCd, which is a six-item questionnaire used to measure nicotine dependence associated with cigarette smoking. It uses a 10-point system, wherein those who scored less than 4 were considered to be minimally dependent, 4–6 were moderately dependent, and 6–10 were highly dependent. FTCd was found to be moderately reliable on an Arabic sample and was valued as 0.68 for Cronbach’s alpha coefficient [41].

We used the validated Arabic version of the ASRS-v1.1, which is a six-item screening tool for ADHD used to assess adult ADHD. It has been proven to be a reliable tool, with a sensitivity of 68.7% and specificity of 99.5%; two-thirds of the clinical cases of ADHD scored 4–6 [44].

## Results

The total number of participants in this study was 506, with 158 (31.23%) males and 348 (68.77%) females. Of the participants, 9.41% were aged between 21 and 24 years, 35.88% were between 25 and 29 years (*P* = 0.007), 44.51% were between 30 and 39 years, and 10.20% were 40 years or older. Of the participants, 46.18% were single, 50.68% were married, and 3.13% were divorced. The majority of the participants (56.19%) did not have any children. The participants were pursuing different majors: 49.32% (majority) were taking courses in humanities/social sciences, 12.72% were studying biological/physical sciences, 12.92% were in engineering fields, and 25.05% were pursuing unspecified majors. With regard to postgraduate studies, 67.72% of the participants were studying for a master’s degree, whereas 32.28% were preparing for their Ph.D.; 26.39% were first-year students, 32.08% second-year students, 20.40% third-year students, 10.30% fourth-year students, and 10.30% fifth-year students. Finally, 33.86% of our participants were studying abroad, whereas 66.14% of the students were studying in their country of origin (Table 1).

**Table 1:** Characteristics of the participants

	Number	%
Gender		
Male	158	31.23
Female	348	68.77
Age		
21–24	48	9.41
25–29	183	35.88
30–39	227	44.51
> =40	52	10.20
Marital status		
Single	236	46.18
Married	259	50.68
Divorced	16	3.13
Number of children		
0	286	56.19
1	67	13.16
2	64	12.57
> =3	92	18.07
Educational level		
Master’s degree	342	67.72
PhD	163	32.28
Academic year		
First	136	26.93
Second	162	32.08
Third	103	20.40
Fourth	52	10.30
Fifth	52	10.30
Being outside the country		
Yes	173	33.86
No	338	66.14

According to the Smartphone Addiction Scale, 51.0% of the participants had high smartphone use, whereas 49.0% had low smartphone use (Table 2).

**Table 2:** Prevalence of Smartphone addiction

Smartphone addiction	Number	%
High smartphone use group	261	51.0
Low smartphone use group	251	49.0

The statistical analysis revealed no significant relationship between smartphone use and the sociodemographic characteristics, such as gender, marital status, number of children, majors, educational level, academic year, studying abroad or in the home country, monthly income, family income, GPA, or number of published papers. However, there was a statistically significant relationship between smartphone use and age (*P* = 0.026). (Table 3).

**Table 3:** Relationship between smartphone addiction and characteristics of the participants

	High smartphone use group (261)		Low smartphone use group (251)		*P*-value
	Number	%	Number	%	
Gender					
Male	88	33.8	70	28.5	0.191
Female	172	66.2	176	71.5	
Age					
21–24	17	6.5	31	12.4	0.026*
25–29	105	40.2	78	31.3	
30–39	117	44.8	110	44.2	
> =40	22	8.4	30	12.0	
Marital status					
Single	115	44.1	121	48.4	0.268
Married	135	51.7	124	49.6	
Divorced	11	4.2	5	2.0	
Number of children					
0	147	56.3	139	56.0	0.867
1	37	14.2	30	12.1	
2	31	11.9	33	13.3	
> =3	46	17.6	46	18.5	
Educational level					
Master	180	69.2	162	66.1	
PhD	80	30.8	83	33.9	
Academic year					
First	80	30.8	56	22.9	0.349
Second	80	30.8	82	33.5	
Third	48	18.5	55	22.4	
Fourth	27	10.4	25	10.2	
Fifth	25	9.6	27	11.0	
Being outside the country					
Yes	94	36.2	79	31.5	0.264
No	166	63.8	172	68.5	
Monthly income					
5000 SR	104	40.3	92	39.3	
5000–10,000 SR	71	27.5	67	28.6	0.952
10,000–20,000 SR	69	26.7	60	25.6	
> 20,000 SR	14	5.4	15	6.4	
Monthly income for father or mother or both					
5000 SR	79	30.5	60	25.1	0.425
5000–10,000 SR	59	22.8	54	22.6	
10,000–20,000 SR	77	29.7	73	30.5	
> 20,000 SR	44	17.0	52	21.8	

In this research, *P*-values < 0.05 were statistically significant. With regard to high smartphone use and age, 35.4% (17) of the participants aged 21–24 years, 57.3% (105) of those aged 25–29 years, 51.5% (117) of those aged 30–39 years, and 42.3% (22) of those 40 years of age or older scored high on the SAS scale (Table 3).

The PHQ-9 for Depression demonstrated a significant association between high smartphone use and MDD (*r* = 0.408) (*P* = 0.001) (Table 4); 65.9% of the participants who were identified to have high smartphone use had no depression, whereas 10.3% had severe depression, 16.1% had moderately severe depression, and 7.7% had moderate depression. Of the non-smartphone addiction group, 81.7% exhibited no depression symptoms, whereas 6.0% showed severe depression, 4.4% moderately severe depression, and 8.0% moderate depression. The multivariate analysis revealed an elevated risk of having severe depression and smartphone addiction simultaneously (OR = 3.779) (*P* = 0.001) (Table 2 and Table 5). In conclusion, high smartphone use is associated with a higher prevalence of depression (Tables 4 and Table 5).

**Table 4:** Relationship between smartphone addiction and smoking, depression, insomnia, and ADHD symptoms

	Smartphone addiction		No smartphone addiction		*P*-value
	Number	%	Number	%	0.039*
Smoking					
Yes	22	8.4	31	12.4	
Low independence	7	2.7	20	8.0	
Low to moderate independence	6	2.3	4	1.6	
Moderate independence	7	2.7	6	2.4	
High independence	2	.8	1	.4	
No smoking	239	91.6	220	87.6	
Depression					
Moderate depression	20	7.7	20	8.0	0.013*
Moderately severe depression	42	16.1	11	4.4	
Severe depression	27	10.3	15	6.0	
No depression	172	65.9	205	81.7	
Insomnia					
insomnia	151	65.7	99	44.4	< 0.001*
No insomnia	79	34.3	124	55.6	
ADHD symptoms					
Have ADHD symptoms	110	47.8	44	19.7	< 0.001*
Have no ADHD symptoms	120	52.2	179	80.3

**Table 5:** Multivariate associations among smoking, depression, insomnia, and ADHD symptoms and smartphone addiction

	Odds ratio	95% CI		*P*-value
		Lower	Upper	
Depression				
Clinically significant depression	1.261	0.562	2.829	0.574
Moderately severe depression	1.43	0.508	4.023	0.498
Severe depression	3.779	1.317	10.846	0.013*
No depression^**^	1			
Insomnia				
Insomnia	2.113	1.372	3.255	0.001*
No insomnia^**^	1			
ADHD symptoms				
Have ADHD symptoms	2.712	1.682	4.374	< 0.001*
Have no ADHD symptoms^**^	1			

* Significant *P*-value

** used as reference

We employed the FTCd to assess nicotine dependence. The total result revealed a moderate significantly positive Pearson’s correlation coefficient between smartphone addiction and smoking (*r* = 0.323) (*P* = 0.018). In our study population, 20.8% were active smokers, and 8.4% of those with smartphone addiction were smokers, indicating that 41.5% of the smokers were addicted to smartphones (*P* = 0.039) (Table 4).

We measured difficulty sleeping based on the AIS; The results demonstrated a significant correlation between the severity of insomnia and smartphone use (*r* = 0.306) (*P* = 0.001) (Table 4); 65.7% of those with high smartphone use had insomnia, whereas 34.3% did not. Conversely, only 44.4% of the non-smartphone addiction group had insomnia, whereas 55.6% were free of it, which indicated a higher prevalence of insomnia among high smartphone users. Smartphone addicts have approximately two times the risk of having insomnia (OR = 2.113) (*P* = 0.013) (Table 5).

We employed the ASRS-v1.1 symptom checklist to consider ADHD symptoms and found that 47.8% of the participants with high smartphone use had ADHD symptoms. Conversely, 19.7% of the non-smartphone addiction group exhibited ADHD symptoms, which indicated a significant relationship between smartphone addiction and adult ADHD symptoms (*r* = 0.405) (*P* = 0.001) (Table 4). Those who had ADHD symptoms were at a greater risk of having smartphone addiction (OR = 2.712) (*P* < 0.001) (Table 5).

## Discussion

Our study demonstrates that 51% of our population scored high on the SAS. A similar study on Lebanese university students employed the Smartphone Addiction Inventory and found that 49% had smartphone addiction [16]. Another study in Saudi Arabia found that 61% of university students had high smartphone use [15].

A significant correlation was observed between age and smartphone addiction. A study in Turkey suggested that gender and young age were correlated with the amount of smartphone use. Specifically, women and younger populations may be at a higher risk for smartphone addiction [28]. However, our results revealed no significant relationship between gender and smartphone addiction. Reaffirming the previous literature, we observed a significantly positive relationship between smartphone addiction and MDD, which was consistently supported by research [25, 32, 33]. In a review of 23 studies, it was found that depression was consistently associated with smartphone use [34]. A study on Korean adolescents observed an association between unhealthy lifestyle habits and smartphone addiction and linked unhealthy diets, weight gain, and sleep disturbance to smartphone addiction. Therefore, these are considered to be symptoms and consequences of MD [35]. A study on university students in Saudi Arabia revealed that 43% of problematic smartphone users had reduced sleeping hours [15]. Our current research indicates that there is a strong association between high smartphone use and insomnia, as most of our subjects (65.7%) reported both. Intensive smartphone use was shown to be positively correlated with poor sleep quality and daytime sleepiness, which was consistent with our findings [36]. Another study conducted at King Abdulaziz University, Jeddah, revealed that mobile use was highly prevalent among participants (73.4% used smartphones > 5 h/day), and two-thirds of the participants had poor sleep quality and latency to sleep [45]. A Belgian study revealed that bedtime smartphone use caused later self-reported rise time, higher insomnia score, and increased fatigue [46]. A study on students between the ages of 18 and 39 indicated that insomnia is associated with high smartphone use [47]. The National Sleep Foundation’s 2011 Sleep in America Poll showed the results indicating that the use of numerous technological devices before bedtime leads to difficulty falling asleep [48]. Confirming our finding of a higher prevalence of ADHD symptoms among students with smartphone addiction (47.8%) compared with low-use smartphone users (19.7%), an epidemiological study employing SAS performed on 4512 South Korean adolescents examined the relationship between smartphone addiction and symptoms of depression, anxiety, and ADHD. It was found that those with smartphone addiction had a higher likelihood of developing ADHD symptoms [49]. Studies found similarities between smartphone addiction and IA [50]. A study that used 12 addiction risk factors to compare smartphone addiction and IA found that there are multiple similar risk factors, such as depression, anxiety, self-control, life satisfaction, and aggression; moreover, the effects of the five identified psychological factors of addiction were all significant (*P* < 0.01) for both IA and smartphone addiction [51]. The current results have revealed a relationship between behavioral addictions and adult ADHD. A previous similar study looked at the relationship between IA and symptoms of ADHD severity and emotional distress through an online survey that established a significant relationship between the severity of IA symptoms and the presence and severity of ADHD symptoms [52]. Furthermore, the studies revealed that individuals with ADHD are more likely to develop other types of behavioral addictions, such as gambling disorders [53, 54]. Adult ADHD was strongly associated with SUD in a literature review of adult ADHD in the Arab world [55].

### Limitations

Due to the nature of cross-sectional studies representing a single point of time rather than a longitudinal observation, it is not guaranteed to be representative of the population. This research cannot be utilized to analyze the behavior of the population over a period of time. Cross-sectional studies do not specify the cause of the disease. There is also a chance of recall bias on the part of participants. Since our study has been circulated online, through emails and various social media channels, it excludes people with MDD, insomnia, or ADHD who do not have access to social media, as well as those who are not interested in taking part in our questionnaire due to social stigmas. Therefore, future research should involve participants who are more open to the idea of mental health and mental illness. In addition, PHQ-9 is the most commonly used questionnaire for the diagnosis of MDD in clinical practice. It addresses somatic symptoms, such as exhaustion and poor appetite, which can be attributed to other diseases, thus placing the study at risk of overestimating MDD prevalence.

## Conclusions

In conclusion, due to the ease of access and utter dependence of smartphones in our daily lives, our mental and physical impacts should be studied across different populations. The postgraduate student population is underrepresented throughout the medical literature. Thus, we hope to expand current knowledge on postgraduate students to include information on smartphone addiction. Confirming several studies, we found a positive association among insomnia, depression, adult ADHD, and smartphone overuse. Therefore, we encourage the scientific community to study the impacts of smartphone addiction on the mental health of postgraduate students. Finally, we recommend that smartphone addiction be carefully monitored in postgraduate students exhibiting depression, insomnia, or ADHD symptoms.

## Data Availability

The datasets analyzed during the current study are available from the corresponding author upon reasonable request.

## Abbreviations

MDD: Major depressive disorder
SUD: Substance use disorders
AUD: Alcohol use disorders
ADHD: Attention-deficit hyperactivity disorder
IRB: Institutional Review Board
SAS: Smartphone Addiction Scale
PHQ9: Patient Health Questionnaire
FTCd: Fagerström Test for Cigarette Dependence Questionnaire
ASRS-v1.1: Adult ADHD Self-Report Scale
IA: Internet addiction
KMO: Kaiser–Meyer–Olkin
